# First Detection and Molecular Characterization of *Colpodella* in Goats, Foxes, and Birds

**DOI:** 10.1007/s11686-024-00959-z

**Published:** 2025-01-24

**Authors:** Kyriacos A. Hasapis, Iris Charalambidou, Catherine O’Dowd Phanis, Stefanie Kazamia, Nicolaos Kassinis, Chad Schou, Panagiotis Karanis

**Affiliations:** 1 Department of Basic and Clinical Sciences, University of Nicosia Medical School, Nicosi, 24005 Cyprus; 2 Department of Life Sciences, School of Life and Health Sciences, University of Nicosia, Nicosia, Cyprus; 3 Game and Fauna Service, Nicosia, Cyprus; 4 Medical Faculty and University Hospital, The University of Cologne, Cologne, 50923 Germany; 5 Department of Basic and Clinical Sciences, University of Nicosia Medical School, 21 Ilia Papakyriakou, 2414 Engomi, P.O. Box 24005, Nicosi, CY-1700 Cyprus

**Keywords:** *Colpodella* sp., Cyprus red fox, Eurasian coot, *Anas* sp., Goats, Cyprus

## Abstract

**Purpose:**

This study aimed to evaluate the prevalence of *Colpodella* sp. in domestic and wild animals in Cyprus. To the authors’ knowledge, this is the first study to report the detection of *Colpodella* sp. in foxes (Cyprus red fox *Vulpes vulpes indutus*), wild birds (Eurasian coot *Fulica atra*, duck *Anas* spp.) and goats (*Capra hircus*) worldwide.

**Methods:**

A total of 180 faecal samples (29 foxes, 48 Eurasian coot, 20 Eurasian teal *Anas crecca*, 7 duck, 44 goats − 10 from a farm and 34 free-living individuals - and 32 sheep *Ovis aries*) were analyzed for the parasite by nested PCR and sequencing.

**Results:**

Four samples were positive (4/180 = 2.2%), including one goat from a farm (1/10 = 10%), one fox (1/29 = 3.4%), one Eurasian coot (1/48 = 2.1%) and one duck (1/7 = 14.3%).

**Conclusion:**

The results of this study support the evidence that *Colpodella* sp. can infect mammals and birds, as well as livestock and wildlife, which could act as zoonotic reservoirs of the parasite and potentially pose a risk to human and animal health.

## Introduction

*Colpodella* are protists that mainly feed on other protists and algae. They are closely related to pathogenic Apicomplexa such as *Cryptosporidium*, *Plasmodium*, and *Toxoplasma gondii*.*Colpodella* sp. are considered to be free-living organisms with a pathogenic capability [1–5]. Recent studies have reported *Colpodella* sp. infections in humans and other mammals. In two cases from China, *Colpodella* sp. caused severe disease in humans. In the first case, *Colpodella* sp. (isolate HEP) infected the red blood cells of an immunocompromised individual causing anaemia [6]. In the second case, the DNA of *Colpodella* sp. (isolate HLJ) was detected in the cerebrospinal fluid of a patient, causing a neurological disorder [7]. *Colpodella* sp. was also incidentally discovered in the urine of a 70-year-old female patient in Romania who was admitted to the hospital for breathing difficulties [8]. Additionally, *Colpodella* infection caused the death of a South China tiger (*Panthera tigris amoyensis)* in a zoo [9]. Reports of *Colpodella* sp. infections in other mammals are limited. *Colpodella* sp. has been isolated from the blood of two horses (*Equus ferus caballus*) (2/400 = 0.5%) in China [10] and one cow (*Bos taurus*) (1/232 = 0.4%) in Zambia [11], faecal samples of captive Felidae (7/56 = 12.5%) at a zoo in China [12] and ear fragments of Racoons (*Procyon lotor*) (3/170 = 1.8%) in Poland [13]. Moreover, two studies indicated that ticks transferred *Colpodella* sp. to the hosts [7, 9]. Another study found a high incidence of *Colpodella* sp. in ticks collected from goat flocks in China [14].

In several studies, *Colpodella* sp. was accidentally detected following PCR amplification with primers specifically designed for *Cryptosporidium* sp. or for other parasites, targeting a part of the *18 S* rRNA gene [10, 12, 14–17]. Similarly, in the present study *Colpodella* sp. was accidentally detected by PCR and sequencing using specific primers designed for the detection of *Cryptosporidium* sp [18–20]. The present study aimed to evaluate the prevalence of *Colpodella* sp. in 180 animal faecal samples by nested PCR and sequencing.

The relatively wide distribution of the genus *Colpodella* sp. and the possibility of infection are a potential public health concern. Here, we provide evidence that this pathogenic protist can infect a great variety of animal hosts, therefore drawing attention to *Colpodella* sp. as an under-reported protist infection in humans, livestock, and wildlife.

## Materials and Methods

### Sample Collection

**Fig. 1:**
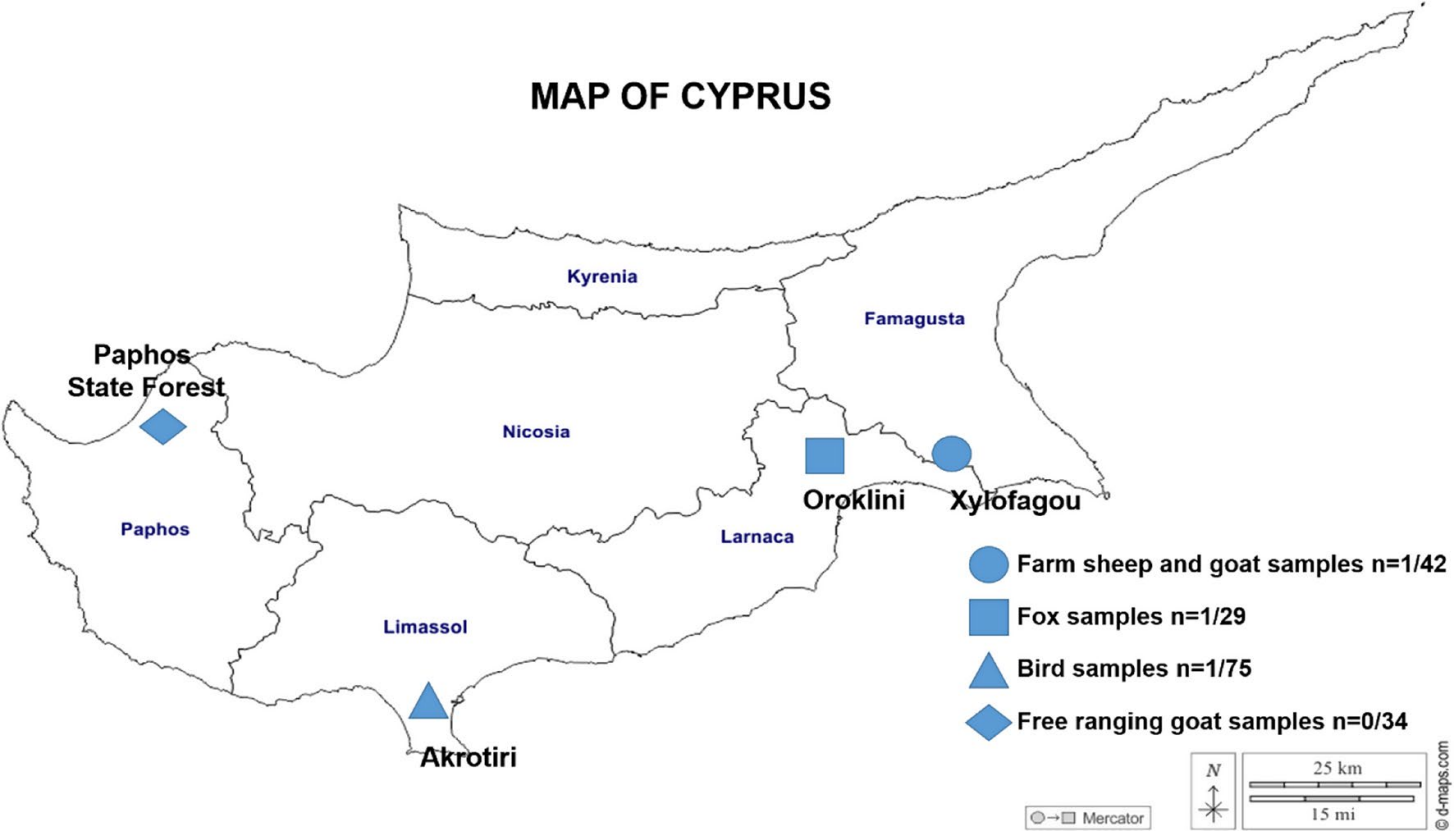
Map of Cyprus indicating the collection sites of the animal faecal samples that were analysed in this study and also the number of positive samples for *Colpodella* sp. and the number of total samples collected per area

One hundred eighty animal faecal samples from livestock and wildlife were collected in Cyprus between January 2021 and April 2023 (Table 1). Specifically, 32 samples were collected from sheep (*Ovis aries*), 10 from goats (*Capra hircus*) at a farm in Famagusta District and 34 from free-ranging goats at Paphos State Forest (Paphos District). At Oroklini Lake (Larnaca District), 29 faecal samples of the Cyprus red fox (*Vulpes vulpes indutus*) were collected. All avian faecal samples were collected from Phassouri reedbeds at Akrotiri wetlands (Limassol District), including 48 Eurasian coot (*Fulica atra)*, 20 Eurasian teal (*Anas crecca)* and 7 duck sp. (*Anas* sp.). All faecal samples were collected from the ground while fresh and placed in sterile 50 ml tubes with animal species/group records, date, location and identification numbers. The samples were transferred to the laboratory on the same day and stored at − 20 °C until DNA extraction within three weeks after collection. A map containing the collection sites for every animal faecal sample is shown in Fig. 1.

**Table 1:** *Colpodella* positives from the analysis of faecal samples of livestock and wildlife in different areas in Cyprus

Area	Host Species name/ Common name	% positive for *Colpodella* spp. (No. positive/ No. sampled)
Farm, Famagusta District	*Ovis aries* Sheep	0 (0/32)
	*Capra hircus* Goat	10 (1/10)
Paphos State Forest, Paphos District	*Capra hircus* Goat (Free-ranging)	0 (0/34)
Oroklini Lake, Larnaca District	*Vulpes vulpes indutus* Cyprus Red fox	3.4 (1/29)
Phassouri Reedbeds, Akrotiri Wetlands, Limassol District	*Fulica atra* Eurasian Coot	2.1 (1/48)
	*Anas crecca* Eurasian Teal	0 (0/20)
	*Anas* spp. Duck	14.3 (1/7)
**Total**		2.2 (4/180)

### DNA Extraction and PCR

According to the manufacturer’s instructions, the total genomic DNA was extracted from the faecal samples using the QIAamp Fast DNA Stool Mini Kit (Qiagen, Hilden, Germany). A nested Polymerase Chain Reaction (PCR) targeting a 600 bp fragment of the *18S* rRNA gene of *Colpodella* sp. was performed. The primers used for the first amplification were SHP1 (forward) 5’-ACC TAT CAG CTT TAG ACG GTA GGG TAT-3’ and SHP2 (reverse) 5’-TTC TCA TAA GGT GCT GAA GGA GTA AGG-3’. The primers used for the second amplification were SHP3 (forward) 5’-ACA GGG AGG TAG TGA CAA GAA ATA ACA-3’ and SSU-R3 (reverse) 5’-AAG GAG TAA GGA ACA ACC TCC A-3’. The conditions used in both amplifications were 94 °C for 3 min, 35 cycles of 94 °C for 45 s, 56 °C for 45 s and 72 °C for 60 s, followed by a final extension of 72 °C for 7 min. For every reaction, a positive and a negative control were used [18].

In this study the PCR primers used for the amplification of the *Colpodella* sp. sequences were originally designed and used for the detection of *Cryptosporidium* sp [18–20]. In the agarose gel electrophoresis analysis, the PCR bands of both *Cryptosporidium* sp. and *Colpodella* sp. have approximately the same size (around 600 bp) and they are equally strong, so the only way to distinguish them is through the sequencing of the purified PCR products. Mixed infection with both parasites in the same animal can be excluded because of the apparent discrimination peaks of the chromatograms of all the sequenced results.

### Sequencing and Phylogenetic Analysis

The bands of the PCR products were gel-extracted from the agarose gel and were purified using the Blirt ExtractMe DNA Kit (Blirt, Gdansk, Poland). The final purified products were sent for sequencing to Macrogen Ltd Europe, Amsterdam (using the SHP3 forward primer of the nested PCR reaction). To identify the species, the sequences obtained from Macrogen were subjected to Nucleotide BLAST (https://blast.ncbi.nlm.nih.gov/Blast) at NCBI GenBank. Furthermore, the sequences were deposited in the NCBI GenBank under the accession numbers OR380968-OR380971. A phylogenetic tree including all the sequences isolated in this study and several reference sequences from the GenBank was constructed using the Neighbor-Joining method by the MEGA 11 software (Bootstrap 1000 replicates). Evolutionary distances were calculated using the Tamura-3 parameter model (https://www.megasoftware.net/*).*

## Results

Four of the 180 faecal samples (4/180 = 2.2%) were positive for *Colpodella* sp. These included one goat from a farm (1/10 = 10%), one fox (1/29 = 3.4%), one Eurasian coot (1/48 = 2.1%) and one duck (1/7 = 14.3%) (Table 1).

**Fig. 2:**
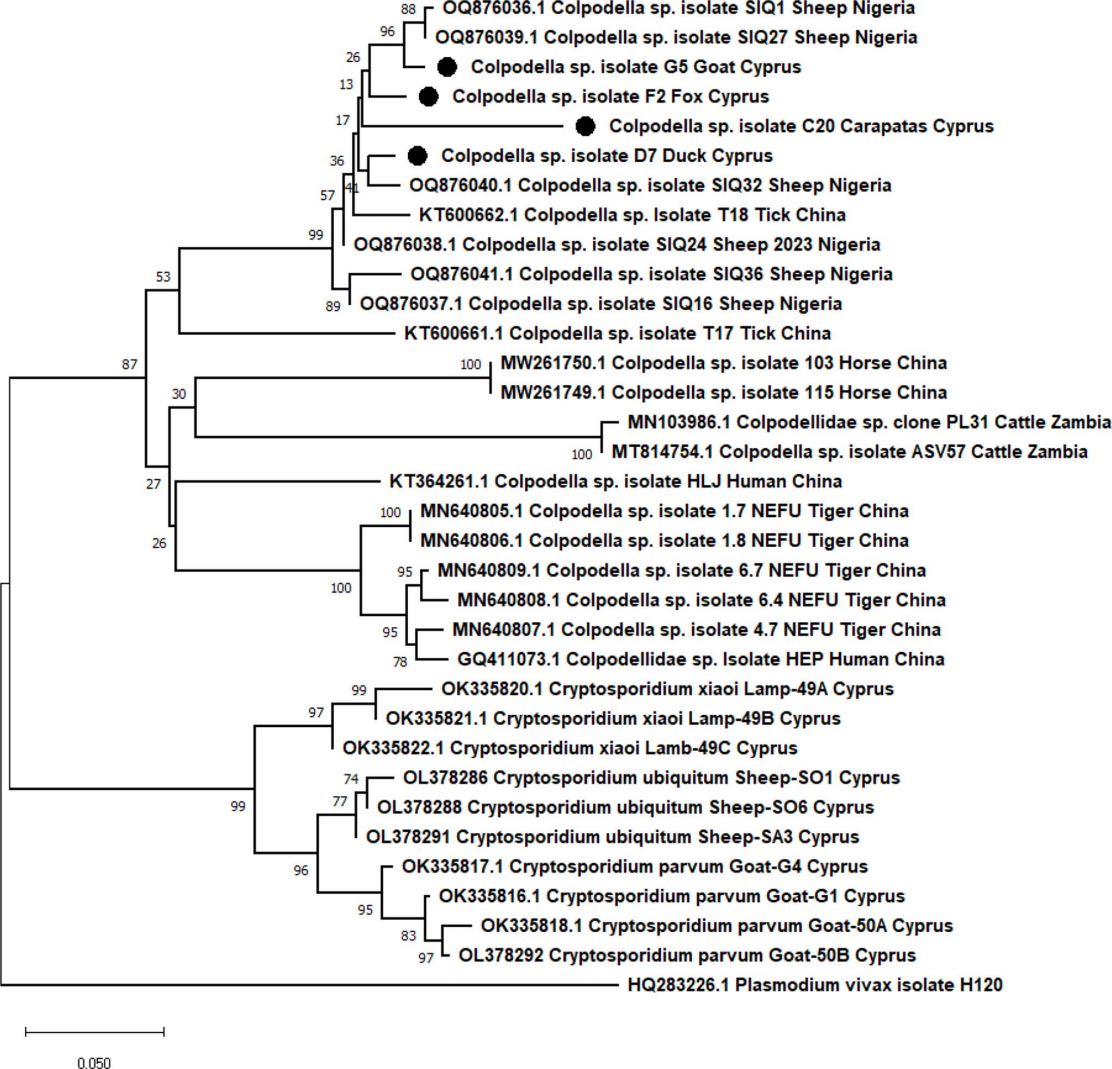
Phylogenetic analysis of *Colpodella* subtypes based on the 600 bp sequence of the *18 S* rRNA gene, using the Neighbor-Joining method with Bootstrap 1000 replicates. Evolutionary distances were calculated using the Tamura-3 parameter model. The percentage of bootstrap samplings is indicated by the numbers above the branches. The phylogenetic tree was constructed using the MEGA 11.0 software. The four *Colpodella* species isolated in this study are marked with a solid circle. Some *Cryptosporidium* species isolated in Cyprus using the same PCR primers as with *Colpodella* sp. (see Hasapis et al. 2023, Schou et al. 2022) are also added in the phylogenetic tree. Finally, *Plasmodium vivax* (HQ283226) *18 S* rRNA gene sequence was used as the outgroup. All the other sequences are reference sequences deposited in Genbank

The validity and elucidation of our results were confirmed after phylogenetic analysis using the Mega11 software. All four *Colpodella* sp. sequences isolated in this study formed a well-defined cluster with every other *Colpodella* sp. sequence (isolated from humans and animals) available in GenBank, including isolates HEP (accession number MH208621) and HLJ (accession number KT364261), as shown in Fig. 2. Interestingly, all four sequences were similar even though they were isolated from different animal species. Also, they all had very high similarity with the *Colpodella* sp. sheep reference sequences from Nigeria, forming a sub-cluster (Fig. 2).

In contrast, the *Cryptosporidium* sp. sequences that were previously isolated by our group [19, 20] formed a distant different cluster despite the fact that they were isolated using the same PCR primers (Fig. 2).

## Discussion

Our findings support previous studies showing that *Colpodella* sp. species are associated with vertebrates. So far, *Colpodella* sp. has been identified in mammalian hosts, including humans [6, 7, 10] felids in China [9, 12] cattle in Zambia [11] and Nigeria (unpublished results, nucleotide sequences found in the GenBank), and racoons in Poland [13]. Additionally, it has been identified in ticks of goats in China [14]. In this study, we have identified goats and foxes as two additional mammalian hosts for *Colpodella* sp., and the positive samples from the bird droppings show for the first time that birds can also host these parasites. This data reinforces the evidence that livestock and wildlife can serve as *Colpodella* sp. reservoirs. More investigations are needed to determine whether a tick vector is involved in the transmission of these avian and mammalian *Colpodella* sp. infections. In addition, the public health risk needs to be evaluated since this protist infection has been under-reported in the literature. Researchers have emphasized the pathogenic importance of *Colpodella* sp. after two case reports of human diseases in China [6, 7], one case report in Romania [8], and the death of a South China tiger in a zoo [9]. Therefore, assessing the zoonotic potential of *Colpodella* sp. infection is crucial for avoiding misdiagnosis in humans and animals [11]. The state of disease due to the *Colpodella* sp. infection has most likely been underestimated, and many case reports have not been documented since there is no established detection protocol for this pathogenic protist.

## Conclusion

This is the first *Colpodella* sp. isolation and molecular characterization report in goats, foxes and birds worldwide. The results of this study contribute to the growing body of evidence suggesting that *Colpodella* sp. may not be a free-living organism and can be pathogenic to its hosts. Therefore, we assume that this organism may be an under-reported potential pathogenic protozoon. Subsequently, there is a need to develop monitoring systems for this protist, and further investigation is required to evaluate the risk of zoonotic transmission in humans and animals.

## Data Availability

No datasets were generated or analysed during the current study.
